# Stem cell transplantation in neurological diseases: improving effectiveness in animal models

**DOI:** 10.3389/fcell.2014.00017

**Published:** 2014-05-14

**Authors:** Raffaella Adami, Giuseppe Scesa, Daniele Bottai

**Affiliations:** Department of Health Science, Faculty of Medicine, University of MilanMilan, Italy

**Keywords:** stem cells, animal models, amyotrophic lateral sclerosis, Parkinson's disease, spinal muscular atrophy, spinal cord injury, epilepsy, stroke

## Abstract

Neurological diseases afflict a growing proportion of the human population. There are two reasons for this: first, the average age of the population (especially in the industrialized world) is increasing, and second, the diagnostic tools to detect these pathologies are now more sophisticated and can be used on a higher percentage of the population. In many cases, neurological disease has a pharmacological treatment which, as in the case of Alzheimer's disease, Parkinson's disease, Epilepsy, and Multiple Sclerosis can reduce the symptoms and slow down the course of the disease but cannot reverse its effects or heal the patient. In the last two decades the transplantation approach, by means of stem cells of different origin, has been suggested for the treatment of neurological diseases. The choice of slightly different animal models and the differences in methods of stem cell preparation make it difficult to compare the results of transplantation experiments. Moreover, the translation of these results into clinical trials with human subjects is difficult and has so far met with little success. This review seeks to discuss the reasons for these difficulties by considering the differences between human and animal cells (including isolation, handling and transplantation) and between the human disease model and the animal disease model.

## Introduction

### Neurological diseases

There are three types of neurological disorders. Firstly there are the disorders which involve a loss of cells in defined subsets of the brain, such as Parkinson's disease (PD), Alzheimer's disease (AD), and Multiple Sclerosis (MS), and Spinal Muscular Atrophy (SMA). Secondly there are diseases where cells are lost following acute damage, such as Stroke, Brain Trauma or Spinal Cord Injury (SCI). Thirdly we have the disorders which involve the impairment of cell function without cell death, like Epilepsy (Leppik et al., 2006) (Table 1).

**Table 1:** **Pharmacological treatments of the common neurological diseases**.

**Pathology**	**Pharmacological treatment**	**References**
Parkinson's disease	Current Parkinson's disease (PD) therapy is essentially symptomatic, and L-Dopa (LD), is the treatment of choice in more advanced stages of the disease. However, motor complications often develop after long-term treatment, and at this point physicians usually prescribe adjuvant therapy with other classes of antiparkinsonian drugs, including dopamine (DA) agonists, anticholinergic, catechol-O-methyl transferase (COMT) or monoamine oxidase (MAO)-B inhibitors	Sozio et al., 2012; Ferreira et al., 2013
Alzheimer's disease	Anticholinergic, inhibitory of NMDA receptor drugs and neuroprotective drugs	van de Glind et al., 2013
Multiple sclerosis	Interferon(IFN)-β and disease modifying drugs	Marta and Giovannoni, 2012; Fernandez et al., 2013
Amyotrophic lateral sclerosis	Riluzole	Morren and Galvez-Jimenez, 2012
Spinal muscular atrophy	No US Food and Drug Administration (FDA) approved treatment for SMA	Cherry and Androphy, 2012
Spinal cord injury	Despite numerous studies reporting some measures of efficacy in the animal literature, there are currently no effective therapies for the treatment of traumatic spinal cord injuries (SCI) in humans. Methylprednisolone (MP) for instance is not FDA approved for this application	Rabchevsky et al., 2011; Hurlbert et al., 2013
Epilepsy	Ca^2+^ channel blockers, GABA uptake inhibitors, Na^+^ channel modulators, GABA_A_ receptor allosteric modulators, NMDA receptor antagonists	Leppik et al., 2006
Stroke	Mostly anticoagulant and thrombolysis agents	Plosker, 2014

A knowledge of the characteristics of the diseases is crucial to finding the appropriate transplantation strategy. Diseases which present, at the time of diagnosis, an extended impairment require an intervention aimed at the replacement of the damaged or dead cells; if the damage is limited, on the other hand, a trophic or anti-inflammatory role for the transplanted cells can be beneficial.

#### Parkinson's disease

When the British doctor James Parkinson, in 1817, described the disorder as “shaking palsy” he was almost 7000 years late. Indeed, the first documented description of this disease is from 5000 BC: in an ancient Indian civilization it was given the name Kampavata. The document which described it recommended treatment using the seeds of a plant containing therapeutic levels of what is today known as levodopa (Manyam and Sanchez-Ramos, 1999).

Parkinson's disease is the second most common neurodegenerative disorder (AD is the most common) and the most common movement disorder. It is characterized by progressive loss of muscle control, which leads to trembling of the head and limbs while at rest, impaired balance, stiffness, and slowness (Jankovic, 2008). Unfortunately, by the time the symptoms are evident, the neurological damage is already severe, with a massive loss of dopaminergic neurons in the *substantia nigra* (Double, 2012).

For over 30 years, the most widely used treatment of PD has been levodopa (L-DOPA) which is converted into dopamine in the dopaminergic neurons by dopa decarboxylase. Since motor symptoms are caused by a deficiency of dopamine in the *substantia nigra*, the administration of L-DOPA pro tempore diminishes the motor symptoms.

Sporadic PD has unknown causes; some hypotheses about the role of environmental toxins were widely supported during much of the 20th century. However, views on the pathology of PD have changed for two reasons, as follow. Firstly, there are no persuasive data to indicate that any specific toxin is a cause of sporadic PD, and chronic environmental exposure to 1-methyl-4-phenyl-1,2,3,6-tetrahydropyridine (MPTP) or rotenone is unlikely to cause PD for chemical reasons (Brown et al., 2006). Secondly, recent work shows that mutation of genes is associated with only a very small proportion of cases (Antony et al., 2013). Only 10% of all PD cases are caused by genetic mutations, and animal models of these mutations (α-synuclein and LRRK2, autosomal dominant PD) and (PINK1/Parkin and DJ-1, autosomal recessive PD) are important since they represent a possible therapeutic target (Dauer and Przedborski, 2003). Most likely the concomitant differences in the genome (for instance the presence of polymorphisms), the patient's age and the presence of environmental factors can all contribute to an increased risk of PD from an epidemiological point of view (Gao and Hong, 2011; Antony et al., 2013). An example of the combination of a variation of the genome and an environmental factor is that an Aldehyde dehydrogenase variation enhances the effect of pesticides associated with Parkinson disease (Fitzmaurice et al., 2014).

#### Alzheimer's disease

More than 35 million people worldwide are affected by AD, a progressive neurodegenerative illness that slowly deprives individuals of their memories and other cognitive functions (Ferri et al., 2005; Sosa-Ortiz et al., 2012). The prevalence of dementia increases from 0.9% in 65- to 69-year-olds to over 30% in people aged 85 years and older (Ferri et al., 2005).

There are many risk factors for dementia, three of which are constant and unchangeable: a family history of dementia, older age, and apolipoprotein E genotype e4 allele. Of the other risk factors for dementia some depend on education and occupational achievements; while others are related to cardiovascular risk factors (smoking, hypertension, diabetes, and obesity) and lifestyle and psychosocial factors (depression, physical activity and alcohol consumption) (Sosa-Ortiz et al., 2012) and can be changed in some ways by the patient.

One of the most important problem is that patients with dementia cannot be healed: the process of cognitive decline can merely be delayed. In numerous countries, cholinesterase inhibitors and memantine are registered for the treatment of cognitive impairment in AD (Cummings et al., 2013).

#### Multiple sclerosis

Multiple Sclerosis is an autoimmune disease which targets the myelinated central nervous system (CNS) tracts. It is the most common chronic inflammatory demyelinating disorder of the CNS, and the leading cause of non-traumatic neurological disability in young adults, affecting 0.1% of the general population in Western countries (Noseworthy et al., 2000). There are many risk factors that could be considered to be responsible for the pathology, among which are environmental risk factors such as infections like measles and Epstein–Barr virus, climate and solar conditions, living conditions and trace elements in the diet (Rosati, 2001).

The inflammation damages the blood–brain barrier and induces the destruction of myelin and the consequent axon damage, gliosis and the formation of sclerotic plaques (Nylander and Hafler, 2012). Continuing lesion formation in MS often leads to physical disability and to cognitive decline. The course of the disease varies between patients, for example more than 60% lose ambulatory capability within 20 years of onset while others are not affected.

Almost 80% of patients will initially present unpredictable attacks (relapses), of variable duration, in which new symptoms appear or existing symptoms become more severe. At the end of the attack, there is a partial or complete recovery. However, symptoms may become more severe and the recovery of function less complete after each attack (Luessi et al., 2012).

No curative therapy is currently available; therapies are mainly directed to preserving CNS cells, inducing remyelination, and modulating T cells. Interferon (IFN)-β was the first agent to show clinical efficacy in the most common form of MS, relapsing-remitting MS. IFN-β treatment reduces relapse rates by about 30%, decreases the formation of inflammatory lesions in the CNS, elongates remission periods, and possibly slows down the progression of disability (Schwid and Panitch, 2007). In determining the risk of developing MS, environmental and hereditary factors need to be regarded as acting in tandem; on this, the lessons learned in connection with PD will assist our understanding of the mechanisms of the pathology for a number of patients (Koch et al., 2013; Munoz-Culla et al., 2013).

#### Amyotrophic lateral sclerosis

Charcot described ALS in 1874. It is the most common form of the neurodegenerative disorders collectively referred to as motor neuron disease, and has a higher incidence in women. The sixth decade is the most common age at which the disease has its onset. 5–10% of cases are familial and the remainder are regarded as sporadic (Rowland and Shneider, 2001). The familial forms involve the mutation of many genes: superoxide dismutase 1 (SOD1) (Rosen et al., 1993), fused in sarcoma (FUS) (Kwiatkowski et al., 2009; Vance et al., 2009), TAR DNA-binding protein 43 (TDP43) (Kabashi et al., 2008), or chromosome 9 open reading frame 72 (C9orf72) genes (Dejesus-Hernandez et al., 2011; Renton et al., 2011). However, a most important recent discovery was that of intronic hexanucleotide repeat expansions in chromosome 9 open reading frame 72 (C9orf72) as a common cause of ALS, frontotemporal lobar degeneration (FTLD) and ALS with concomitant FTLD. The high frequency of C9orf72 mutations in patients lacking a family history of ALS further blurred the distinction between the sporadic and the familial forms of ALS and FTLD (Dejesus-Hernandez et al., 2011; Renton et al., 2011).

Clinical studies had already suggested that ALS and FTLD represent two extremes of a spectrum of neurodegenerative disorders, which co-occur in up to 15% of patients (Lomen-Hoerth et al., 2003). An association between dementia and ALS was noticed as early as the late 19th century. After this initial discovery, many other authors reported similar data (Neary et al., 2000).

The strongest evidence for considering ALS and FTLD as one disease, however, comes from the discovery that C9orf72 mutations are a prevalent cause of ALS, ALS-FTLD, and FTLD (Kwiatkowski et al., 2009; Vance et al., 2009) and are most likely responsible for approximately 40% of ALS and for approximately 25% of familial FTLD (Van Blitterswijk et al., 2012).

ALS is a heterogeneous disorder at almost all levels: clinical, genetic and mechanistic. For instance the ranges of age of onset and rate of progression of the disease are wide; even when the same type of mutation is present, age at onset and survival rates vary substantially (Regal et al., 2006). This heterogeneity remains unexplored and little understood (Van Damme et al., 2013).

Drugs have little effect on disease progression, so other, multidisciplinary, strategies are required. For instance artificial ventilation and feeding tubes are the main care options. Furthermore, since cognitive functions and emotional ability seem to be affected by this disease (Abrahams et al., 1995) psychological intervention is desirable.

The only drug approved by the U.S. Food and Drug Administration for the treatment of ALS is riluzole, a glutamate antagonist. In two therapeutic trials, riluzole prolonged survival by 3–6 months (Bensimon et al., 1994; Lacomblez et al., 1996).

#### Stroke

Stroke is the abrupt loss of brain function due to alteration in the blood supply to the brain. It is recognized as the second leading cause of death worldwide; its incidence depends on age and race (Grossman and Broderick, 2013).

Small and large artery occlusions are the main factors responsible for the pathology, with the occlusion of intra- and extracranial large vessels seeming to involve endothelial injury and platelet aggregation. When smaller vessels are occluded there is a similarity with arteriosclerosis due to common vascular risk factors of diabetes, hypertension, and hypercholesterolemia. The canonical therapeutical approach is mostly directed to the reduction of the thrombus and the prevention of clot formation (Grossman and Broderick, 2013; Plosker, 2014). Little is known and little action is taken of a neuroprotective or neuromodulatory nature on patients affected by stroke. The main target is the recovery of language by means of dopamine precursors, or agonists, or cholinergic neuromodulation (Breitenstein et al., 2006).

#### Spinal muscular atrophy

Spinal muscular atrophy is one of the most devastating childhood diseases since it affects babies from birth onwards (it can occasionally be detected during gestation), and in its more severe form—type 1 or Werdnig-Hoffmann disease, in which patients cannot sit and some of them cannot control the position of their head—life expectancy does not exceed 2 years. Type 2 SMA is an intermediate form whose onset is between 7 and 18 months of age; patients can sit but never stand and they can survive to adulthood. Type 3 SMA (Kugelberg and Welander) has its onset after the 30th month of life. The severity of the disease is classified by the degree of muscle weakness (before or after 3 years); the patient can walk but in some more severe forms they stop walking in adulthood. Finally type 4 SMA has its onset between the 10 and the 30th years of life; length of life is as with type 3 and patients can stand and walk and—if well trained—continue doing so all their life (Mercuri et al., 2012; Bottai and Adami, 2013).

SMA is a genetic disease caused by a loss of function mutation of a telomeric gene called Survival Motor Neuron 1 (SMN1) (Burglen et al., 1995, 1996). The pathology is very variable and depends on the number of copies of another centromeric gene, the Survival Motor Neuron 2 (SMN2), which can transcribe for the same protein although with a lower rate of expression (Campbell et al., 1997; Bottai and Adami, 2013). So far no pharmacological treatment has been shown to be effective, although the various clinical trials performed even recently need to be revisited, as there is a great variability of response to pharmacological treatment between different patients (Garbes et al., 2013).

#### Spinal cord injury

Mechanical damage to the spinal cord results in dramatic change in the capabilities of the CNS. In SCI the force applied to the bones (due to work, or car, bike or sports accident) can deform and break the bone itself and can damage the nervous tissue.

This is called primary damage and depends on the amount of energy transferred to the nervous tissue. In this pathology there is, however, secondary damage due to the response of the immune system of the patient, which tries to repair the damage but actually causes more impairment. Indeed, many substances produced by leukocytes are neurotoxic and have been implicated in the onset and progression of CNS autoimmune and neurodegenerative diseases (Feuerstein et al., 1998). The secondary damage in many cases is even more severe than the primary damage, and is often responsible for the chronic effects that the patient will face in later life.

Due to the wide range of degree and type of damage in human patients it is difficult to find a single strategy for the treatment of SCI: in most cases the current therapeutic algorithm includes early surgery consisting of decompression of the spinal cord and stabilization of the spine in indicated cases. As soon as the patient's injuries (often multiple) become stable, the patient is transferred to a specialized rehabilitation center. Nonetheless, there is no treatment available today that can lead to the repair of the damaged spinal cord tissue. The current standard therapy consists of the administration of methylprednisolone sodium succinate (MPSS) to reduce SCI damage by decreasing lipid peroxidation and free radical production, and preventing edema taking place during ischemia and re-perfusion.

#### Epilepsy

Epilepsy, also known as seizure disorder, is a pathological condition that brings about seizures and affects a range of mental and physical functions (Mattson, 2003). Seizures are caused by a malfunction of the electrical system of the brain, with an uncontrolled discharge that makes the brain cells keep firing. This results in a flux of energy through the brain, causing muscle contractions and unconsciousness. When a person has at least two seizures without another known cause, they are considered to have epilepsy.

There are various kinds of seizures, which the experts divide into generalized seizures (absence, atonic, tonic-clonic and myoclonic), partial (simple and complex) and status epilepticus (Mattson, 2003; Beydoun and D'souza, 2012).

In many cases—about 70%—no cause can be found. In other cases, the epilepsy can be due to head injuries or lack of oxygen during parturition, which may alter the delicate electrical system in the brain, genetic conditions (such as tuberous sclerosis), lead poisoning, brain tumors, problems in the development of the brain before birth and infections like meningitis or encephalitis.

Anti-epileptic drugs vary in structure and function, and in many cases their clinical activity is not understood. The anti-epileptics have three main intended effects: membrane stabilization, reduction of neurotransmitter release and increase of GABA-mediated inhibition (Leppik et al., 2006; Howard et al., 2011).

### Transplantation methods

Transplanting cells involves different sets of questions that need to be taken into account in preclinical and clinical trials.

#### Disease

The first one is whether the pathology induces the death of brain cells or rather initiates a change in the interactions between cells. The second concerns the possibility—which exists only when it is known that the blood brain barrier (BBB) is open—of systemic transplantation. A further one, with regard to the disease itself, is whether the pathology induces an inflammatory response, in which case the role of the transplanted cells should be not only substitutional but also anti-inflammatory. These are all relevant concerns needing to be taken into account in the case of almost all the cells that have been used in clinical trials.

#### Stem cell use

Given the foregoing, the choice of the cells to be transplanted can be very wide.

Many different types of stem cells have a potential therapeutic role in the treatment of neurological diseases (Table 2). We can divide the approach into two large sections, according to the role that the transplanted cells are supposed to play: substitutional and trophic.

**Table 2:** **Stem cells used for the transplantation in neurological diseases**.

**Stem cell type**	**Origin**	**References**
Embryonic stem cells	Inner cell mass of the blastocys	Evans and Kaufman, 1981; Thomson et al., 1998
Induced pluripotent stem cells	Reprogrammed adult tissue cells	Takahashi and Yamanaka, 2006
Mesenchymal stem cells (including Amniotic fluid stem cells)	Many different tissues: bone, fat, cartilage, stromal cells of the bone marrow, and fetal appendages	De Coppi et al., 2007; Nagai et al., 2007; Bottai et al., 2012; Frenette et al., 2013
Neural stem cells	Human fetus	Weiss et al., 1996; Vescovi et al., 1999b
Muscle stem cells	Skeletal muscle	Cooper et al., 2006

In the early days of stem cell transplantation in neurological diseases the substitutional role was the focus of hypotheses and much optimism. Many scientific works in animal models have shown that transplanted cells lodging in the nervous tissue were not sufficient to exert any effect or to bring about any physiological outcome (Pluchino et al., 2003, 2009; Bottai et al., 2008; Cusimano et al., 2012; Nizzardo et al., 2013).

If the therapeutical approach is adopted when the neurological impairment is already substantial it is very difficult to reconstitute the tissue and, therefore, to rebuild the damaged neural circuits. Indeed, injury to the spinal cord involves the loss of motor-neurons with long axons that are surrounded by myelin sheets. In these cases the transplanted cells have to reconstitute not only the neurons but also the glia; moreover, they need to be able to extend their processes in the right direction in order to exert their therapeutic action.

With current knowledge it is unlikely that this task could be achieved but the combination of transplanting therapies and bio-engineering (constructing scaffolds) could be a new avenue for transplantation research in finely structured tissues.

## Different types of stem cells used for the treatment of neurological diseases

From a physiological point of view we can divide stem cells into embryonic, fetal and adult cells (Table 2). The differences between them are in their origins, their proliferation and differentiation capabilities and their telomere stability.

### Embryonic stem cells (ESCs) and induced pluripotent stem cells (iPS)

Since the early 1980's (Evans and Kaufman, 1981) it has been well known that ESCs possess high proliferation and differentiation capabilities and were able to generate a whole mouse (Nagy et al., 1993). In the late nineties the possibility of producing human ESCs was also demonstrated (Thomson et al., 1998). Unlike normal somatic cells, ESCs do not face senescence and can be grown in virtually unlimited quantities, retaining high telomerase activity and normal cell cycle signaling.

ESCs have been used for many years in different models of neurodegenerative diseases. For instance, in 2002 Isacson demonstrated that mouse undifferentiated ESCs transplanted into the striatum of a rat model of PD resulted in the differentiation of Dopaminergic (DA) neurons and caused sustained behavioral restoration of motor asymmetry (Bjorklund et al., 2002). A few years later, two groups demonstrated that primate ESCs differentiated *in vitro* were able to induce a partial recovery in parkinsonian monkeys (Takagi et al., 2005) and rats (Ferrari et al., 2006) and were able to integrate in the striatum, generating Tyrosine Hydroxylase (TH)+ neurons. Also SCI has been treated using the transplantation of ESCs either using differentiated ESCs (such as oligodendrocytes precursors) (Liu et al., 2000), where the cells migrate and differentiate in mature oligodendrocytes capable of myelinating axons or undifferentiated cells (Bottai et al., 2010) where they have mainly a trophic role, reducing the inflammation and preserving the myelin of the ventral columns.

Retinoic acid pretreated ESCs were also successfully used in ischemic rat models (Wei et al., 2005) where they enhanced functional recovery on neurological and behavioral tests. Moreover, motor neuron differentiated ESCs were able to induce a motor improvement in a genetic rat model of ALS (Lopez-Gonzalez et al., 2009), and multipotent neural precursors (NPs) reduced the clinical signs of MS in a mouse model of experimental autoimmune encephalomyelitis by means of the attenuation of the inflammatory process (Aharonowiz et al., 2008).

Regardless of their potentiality the use of undifferentiated ESCs raises considerable numbers of concerns about the formation of tumors and teratomas, although such a risk decreases with their progressive cellular differentiation (i.e., reduced multipotency); in addition to these factors, we must not forget that there are many ethical concerns around ESCs.

In 2006 a new frontier was opened up by Yamanaka (Takahashi and Yamanaka, 2006). The production of embryonic-like stem cells originating from adult cells (mostly fibroblasts) put an end to the ethical concerns around the use of pluripotent stem cells. These induced pluripotent stem cells, obtained by the introduction of four genes Oct3/4, Sox2, c-Myc, and Klf4, which have a transcriptional factor activity in the early phases of their development, have physiological and molecular characteristics similar to ES with respect to their proliferation and differentiation potentiality. Moreover, *in vivo* iPS induction in mice demonstrated that in experimental conditions the iPS have an unexpected capacity to form embryo-like structures including the three germ layers and the extra-embryonic structures, indicating that induction *in vivo* can achieve an even earlier stage of development than the ESCs (Abad et al., 2013).

The affinity of iPS with the ESCs makes these cells suitable for a similar application in animal models of neurological pathology. Indeed, it has been demonstrated that human iPS differentiate into DA progenitor cells and transplanted into a chemically induced PD rat survive long term and develop into DA neurons and integrate into the brain parenchyma. However, some cells produced tumour-like nestin positive cells, raising some concern about the safety of these cells (Cai et al., 2010); indeed, in another study, in order to minimize the risk of tumour formation the dopaminergic derived iPS cells were separated from contaminating pluripotent cells by means of fluorescence-activated cell sorting (Wernig et al., 2008). Protein-based iPS differentiated to the terminally-matured DA neurons as the ESCs did, but had higher levels of DA neuron-specific markers' expression than ES cells, indicating that iPS were a suitable source for PD patient-specific treatment (Kwon et al., 2014).

Similarly, neuroepithelial-like stem cells from human iPS cells were used to treat SCI in mouse. In this model they were able to differentiate into neural lineage and cause a recovery of motor function (Fujimoto et al., 2012; Kobayashi et al., 2012).

Ischemia induced by middle cerebral artery occlusion was treated by means of astroglial- and neuron-like differentiated iPS using a fibrin glue support. iPS cells were able to improve the motor function, attenuate inflammation, reduce infarct size and mediate neuroprotection in this model (Chen et al., 2010).

Concerning the fetal and adult stem cells obtained from differentiated tissue in the fetus and in mature organisms, many different types of cells can be described that have some (at least preclinical) applications.

### Neural stem cells

The telencephalon and the diencephalon of the human fetus between the 9.5 and the 12th weeks of gestation possess cells with all the characteristics of stem cells. They proliferate at a ratio that could allow transplantation into human patients to treat various pathologies and can differentiate into neurons (that have physiological electrical activity), astrocytes and oligodendrocytes (Vescovi et al., 1999a) in a similar way to how neural stem cells obtained from rodents do so (Gritti et al., 1999; Bottai et al., 2003).

Neural stem cells have been successfully applied to many different animal models of neurological diseases. They were used in an MS mouse model and induced either a substitutional effect, especially at the level of the oligodendrocytes, which were able to reconstitute the myelin sheets, or a trophic effect by means of the production of different cytokines (Pluchino et al., 2003).

The same cells were also intravenously transplanted into a mouse model of SCI and they had no capacity to rebuild the damaged tissue but they were able to reduce the inflammation through the production of neurotrophic factors (Bottai et al., 2008), indicating that the response of the same cells differs according to the pathology. Analogous results were obtained via intraspinal transplantation of neural stem cells, and in this case an immunomodulation was also obtained (Cusimano et al., 2012). On the other hand, human neural stem cells transplanted into an SOD1 rat model of ALS by means of multiple segments in the spinal cord injection have been shown to ameliorate the disease, delaying the onset and prolonging survival (Xu et al., 2011). In addition, human neural stem cells were used for the treatment of ischemia in adult rats, where they migrate and differentiate in the rat brain with focal ischemia and improve functional recovery (Chu et al., 2004b).

### Mesenchymal stem cells (MSCs)

Mesenchymal stem cells can be retrieved from various adult tissues such as fat, cartilage, stromal cells of the bone marrow, dental pulp, skin, and from fetal appendages (De Coppi et al., 2007; Bottai et al., 2012; Moroni and Fornasari, 2013). MSCs have many disadvantages (relative to ESCs or iPS) such as insufficient numbers of stem cells, reduced proliferation and differentiation capacity with age *in vitro* and after stem cell transplantation *in vivo* (Rao and Mattson, 2001). However, to date, no evidence of spontaneous transformation have been described; as a matter of fact a study which reported such an event published in 2005 (Rubio et al., 2005) was retracted in 2010 (De La Fuente et al., 2010).

These data prompted researchers to find other sources of MSC, and the search was directed to fetal tissues and in particular fetal appendages such as cord blood (Malgieri et al., 2010), amniotic fluid (De Coppi et al., 2007; Bottai et al., 2012) and placenta (Zhu et al., 2013). Results of flow cytometry revealed that cells isolated from human umbilical cords, amniotic fluid and placenta expressed CD29, CD44, CD73, CD90, and CD105, but not hematopoietic- or endothelial-specific antigens CD14, CD34, CD45, CD 106, CD 133, or HLA-DR (MHC-II) (De Coppi et al., 2007; Bottai et al., 2012; Zhu et al., 2013).

These cells can probably not have a substitutional role in neurological diseases; indeed, in many examples their contribution is mainly one of immunomodulation. The work performed by Uccelli's group is enlightening. This author has described in many papers the roles of MSCs in two neurological pathologies: MS and ALS (Lanza et al., 2009; Morando et al., 2012; Uccelli et al., 2012). The main outcome of these paper was that MSCs exert their effects by means of antioxidant and neuroprotective activity. Similar results were described in a mouse model of SCI where systemic treatment with Amniotic Fluid Stem cells (AFCs) was able to induce some recovery of motor function and partial spinal cord tissue preservation through an anti inflammatory mechanism which involved the production of the hepatocyte growth factor (Bottai et al., 2014).

## How efficiently can transplantation in animal models be translated into use in treating human diseases?

One major issue common to all types of disease is the validity of any single model or group of models (McGonigle, 2013). One attempt to provide a rigorous mode of assessment was the set of criteria proposed by Willner (1984) for use in the evaluation of an animal model for CNS disorders. In order to study a human pathology the choice of an appropriate animal model for preclinical study is required since it will allow a more feasible translation to clinical study. An ideal animal model, will have many attributes: a comparable anatomy and physiology; a similar genetic basis; close pathological response(s) and underlying mechanism(s); a phenotypic final stage similar to clinical studies; responsiveness to known drugs with clinical efficacy and predictiveness of clinical efficacy. Even when the animal model fulfills all of these criteria, in many cases translation into human trials results in difficulties or poor success rates. An understanding of the reasons why this transposition is unproductive is therefore necessary but unfortunately it has to be adapted model by model and pathology by pathology.

When a new chemical entity (NCE) is introduced for the treatment of a pathology an appropriate study of the pharmacodynamic/pharmacokinetic (PD/PK) relationships is necessary (Fan and De Lannoy, 2013). These steps are already difficult with conventional drugs since in many cases the animal model has significantly different pharmacokinetics; moreover, in many cases the pathology is not present in nature for the animal model so there can be also significant differences in the pharmacodynamics, especially in the case of transgenic and knockdown models. All these points are exponentially magnified when we are faced with a cellular approach to the pharmacologic treatment.

### Variations in the transplantation approach

The first issue to be taken into account in the animal model used for the pathology is of course that it has to be as close as possible to the human counterpart, but there are pathologies that do not exist in animals in nature. One example is that SMA is not present in the mouse, which has only one smn1 gene and when this is missing or is mutated this condition is not compatible with life; another example is that, monkeys have many copies of Smn1 and if one is not functional there is no appreciable effect on the animals (Bottai and Adami, 2013). So the use of these models by scientists has to be evaluated very carefully.

Another important point concerns the type of cells that should be used in the pathology. If, for example, the aim is to assess the efficacy of stem cell treatment in a mouse model of SCI, it would be wise to use mouse stem cells in order to avoid the need for immuno suppression treatment. In this case the translation to clinical trial will necessitate verification of the characteristics of the human cells, and, in many cases, their use in preclinical studies before starting phase 1 of clinical trial.

In view of this, it seems necessary to use human stem cells from a range of sources in animal models of neurodegenerative pathology. Consequently in many situations the animal model of the human pathology needs to be treated with immunosuppressive agents in order to avoid the rejection of the transplanted cells; thus, implicitly, a new variable is introduced into the experiment and the analysis will be much more convoluted. On the other hand, some types of cells such as MSC are able to exert immunomodulatory effects and could be transplanted without immunosuppressive treatment of the animal (Dazzi et al., 2012).

### Differences in the preparation of the cells

Initiating the clinical study brings the need to work according to Good Manufacturing Practice conditions (GMP). At this stage a large number of new culturing and transplanting settings must be introduced, which will include the appropriate laboratory facilities and the appropriate materials that are needed to achieve “human standard.”

Such steps result in a very large increase in the costs of the clinical trial but they are necessary to ensure the safety of the treatments in humans. Moreover these changes in mode of preparation can, in some situations, interfere with the properties of the cells and reduce their usefulness for transplantation.

Another factor that needs to be taken in account is the mode of transplantation adopted, for instance, whether it is performed locally, regionally or systemically. This decision involves many different questions, such as how many transplantations could be performed, how many cells need to be transplanted and, consequently, how many cells must be cultivated. This last question—the number of cells needed—is of particular interest when considering the step between the preclinical and the clinical trial. While, for instance, we use 10^6^ neural stem cells to transplant a spinal cord injured mice (transplantation in the tail vein) (Bottai et al., 2008), for a human we will need many more cells due to the human's body weight being roughly 2000 times greater. This is quite apart from the different pharmacokinetic properties of the human organism relative to the animal; indeed, the metabolism of the mouse is much higher than the human's. For these reasons a more regional transplantation is desirable.

### Standard procedures for transplantation approaches

**Figure 1:**
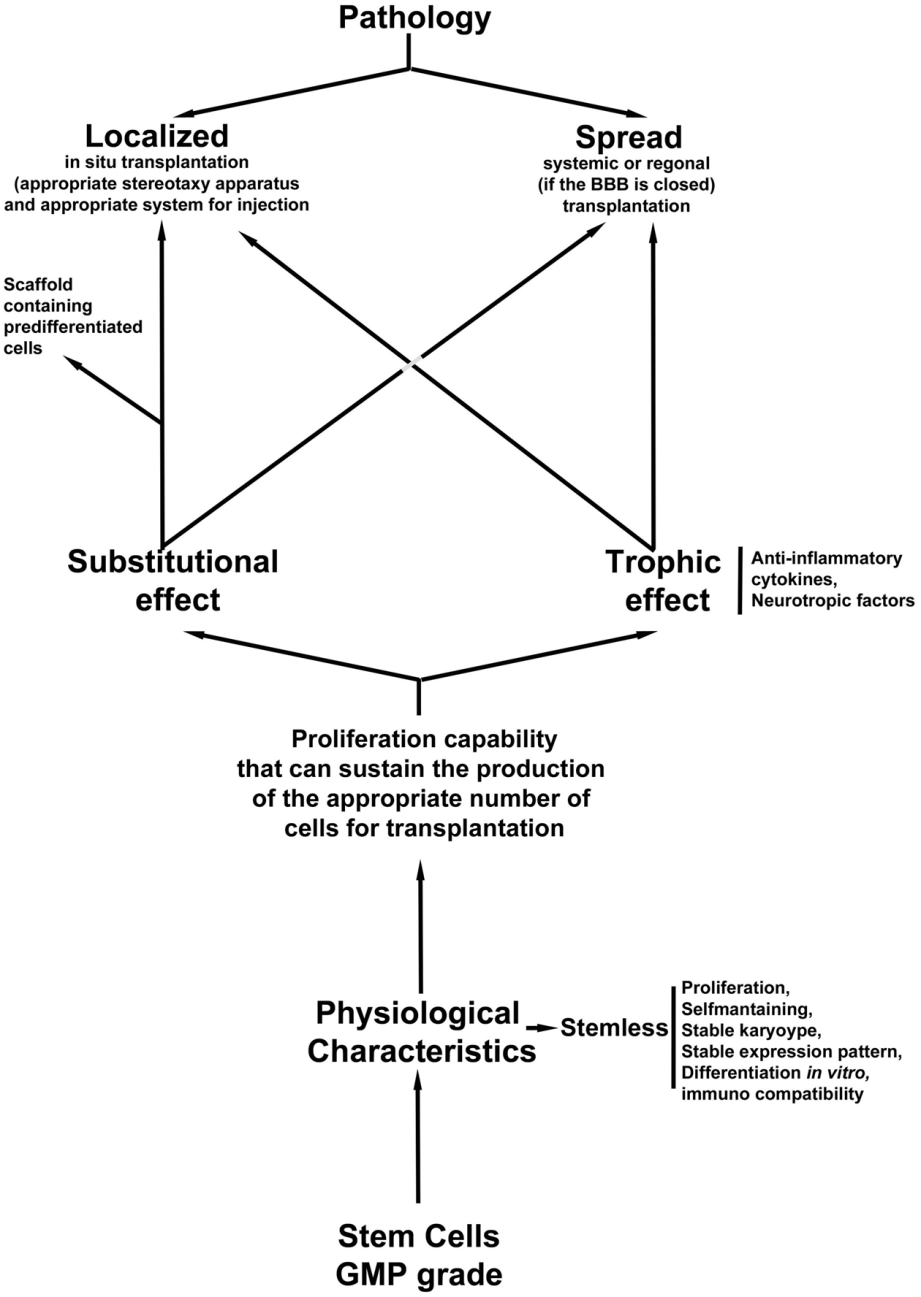
**Flow chart of the appropriate decisions for the choice of stem cells and the method of transplantation**.

In summary, the choice of cells and the mode of transplantation adopted are both crucial for the successful outcome of the treatment. The diagram in Figure 1 shows a flow chart that could be followed in order to make appropriate decisions for the choice of stem cells and the method of transplantation.

In this scenario, a quality control step is needed: cells must be GMP grade; however, the analysis of their safety vis-à-vis the stability of the culture is mandatory. A karyotype, or-better- an expression panel of the cells is needed before transplantation in order to check whether the culture is stable after various passages *in vitro*. Moreover, in order to clarify the choice of strategy, we need to evaluate whether the cells are able to differentiate and substituted the damaged cells (if we plan to have a substitutional approach), or if they can produce growth factors (if we believe that the best approach for the pathology is a trophic intervention). In some cases, localized damage could be treated in a systemic fashion as was demonstrated in an SCI animal model (Bottai et al., 2008, 2010, 2014) where the role of the cells is mostly trophic. A third option of the transplantation strategy is the use of support scaffolds that could sustain an appropriate growth of pre differentiated stem cells if the damage is localized such as in the SCI. In this case the scaffold needs to be able to support controlled proliferation, differentiation and maturation.

Regarding the pathology, we first need to decide whether the intervention will be localized with an *in situ* transplantation or it will be systemic or regional. In an *in situ* delivery of the cells an appropriate stereotaxy apparatus will be needed (such as computed tomography (CT−) or Magnetic resonance imaging (MRI)-guided stereotaxic neurosurgery Brundin et al., 2000) in order to perform an injection in the correct three dimensional position, as is required for instance in transplantation in PD patients or in spinal cord injured people. On the other hand, if the pathology has affected many different sections of the CNS, a systemic or (if the BBB is not open) regional intervention could be appropriate.

### Stem cell protocols

As mentioned above many factors need to be taken into account in dealing with the transplantation of stem cells in a pathology and in particular in a neurodegerative disease. The following section offers a description of the parameters that we need follow when stem cells are prepared before the transplantation, with MSCs being used as a representative example. Meanwhile we have to keep in mind that, as pharmaceutical tools stem cells are very stringently regulated: in Europe is ruled by the regulation (EC) No. 1394/2007 on Advanced Therapy Medicinal Products (ATMPs), which lays down specific guidelines concerning supervision, pharmacovigilance and centralized authorization (Martins et al., 2014).

### Isolation of MSCs

Because MSCs are spread widely through the human body, several different procedures can be adopted for their isolation.

Mesenchymal stem cells can be isolated from many different tissues such as fat, cartilage, stromal cells of the bone marrow, dental pulp and skin and from fetal appendages (De Girolamo et al., 2013; Moroni and Fornasari, 2013; Ikebe and Suzuki, 2014); and different strategies must be used to extract the MSCs according to which tissue is used.

For instance, the ATMPs certification is required in order to meet the guideline criteria of the International Society for Cellular Therapy (ISCT), so a specific isolation protocol for umbilical cord tissue was patented.

The procedure for bone marrow MSCs preparation comprises three steps: (a) Cells undergo an initial decontamination step using an efficient antibiotic/antimycotic, (b) All the enzymes used are clinical grade and fetal bovine serum is substituted by non-animal materials, and (c) the absence of mycoplasma- and endotoxins absences is ensured by using appropriate sample handling materials and cell culture reagents (Martins et al., 2014).

Additional, tissue dependent, steps are necessary; for example, the amounts of enzymes and cofactors needed for the dissociation of the tissue must be optimized. The first seeding steps will be performed in a non-animal free serum condition and in a static horizontal monolayer in order to allow the elimination, the following day, of the non-adherent cells, which are discarded and the medium changed. When the ATMP-adapted protocols is required the first step will be the use of plastic disposable ware and all sample handling material and cell culture materials need to be certified as mycoplasma-free and as having an acceptable low endotoxin level. Moreover, the culture must to be monitored on a regular basis for the visible detection of bacterial or fungal contaminations and for mycoplasma and endotoxin contamination using the appropriate detection kit. The ATMP cultures need to be compared with those produced under standard non GMP protocols in order to verify that their characteristics are maintained in these new conditions; for this purpose an Affymetrix GeneChip analysis on 47,000 human transcripts is desirable (Martins et al., 2014).

Another important aspect that needs attention during the preparation of cultures of ATMP grade is the flow cytometer immune phenotypic analyses. For cord blood MSC cells, the surface markers used are CD44; CD73; CD90; CD14; CD45; CD31; CD34; CD19; HLA-DR, and CD105 (Martins et al., 2014).

Two further keys points in testing of the ATMP grade of the cells are the evaluations of their differentiation capability and of their teratoma-forming potential. Adipogenic, chondrogenic and osteogenic differentiations are performed in a standardized fashion following already established protocols (Santos et al., 2013). The teratoma assay formation is performed in immunodeficient male, C.B.-17/GbmsTac-scid-bgDF N7 mice (6 weeks old), using the candidate MSCs and the ESCs H9 as positive control, implanting 1 × 10^4^ cells beneath the testicular capsule; the teratoma growth is analyzed 6.5–8.5 weeks post implantation after the sacrifice of the animal (Martins et al., 2014).

A final issue which needs particular attention for the handling of MSC cells from cord blood, but which is also relevant to all stem cells, is cryopreservation.

This procedure usually involves slow cooling in the presence of a cryoprotectant to avoid the damaging effects of intracellular ice formation. 1–2°C/min and rapid thawing is considered standard, whereas the passive cooling devices which employ mechanical refrigerators, generally at −80°C, do not offer sufficient reproducibility for the ATMP-grade cells'.

Dimethyl sulphoxide (DMSO), is the most widely used cryoprotectant, but it is known to be toxic at certain temperatures, times, and concentrations to stem cells and tissue, especially if the transplanted cells are not cleaned of it before transplantation. For these reasons polyvinylpyrrolidone (PVP) has been used in order to reduce the concentration of DMSO (Hunt, 2011). In this context, the response in terms of the maintenance of stem cell characteristics depends on the type of stem cells. For example hESCs are more sensitive to conventional cooling than MSCs, with a lower recovery (16% of viable cells after freezing and thawing) relative to MSCs and with a lower size of colonies and a significant degree of differentiation relative to the cells that had not undergone cryopreservation (Hunt, 2011).

For the cryopreservation of MSCs from bone marrow in particular the standardized number of stored cells per vial is normally 3 × 10^6^. These cells have to be centrifuged at appropriate speed, then resuspended in the AMTP appropriate cryobuffer (such as UCX®-ATMP in Biofreeze (Biochrome) and frozen by means of a Controlled Rate Freezer (CRF) (IceCube14S, Sylab) (Martins et al., 2014). The maintenance of the cell can be pursued in N2 fumes at the temperature of −135°C and a specific freezing profile (Freimark et al., 2011).

A note on speeds: 200 g is optimal for cord blood stem cells but for different kinds of cells the speed needs to be determined, for instance NSCs that are grown in suspension as neurospheres need a lower centrifugal force to be pelleted.

Finally, a new procedure for cryopreservation is vitrification. During conventional slow cooling, ice formation and an increase of solute concentration are responsible for damage to the cells. DMSO is able to reduce such damage by reducing of the amount of ice formed. In vitrification the cryoprotectants are at a concentration that completely avoids the formation of ice crystals. This is achieved by the high concentrations of solutes and/or by rapid cooling. While cooling continues, viscosity increases until all molecular motion comes to halt and the solution becomes a glass, displaying the properties of a solid but retain the molecular structure of a liquid (Hunt, 2011). This method is particularly suitable for hESCs, allowing them to conserve their properties.

## Use of stem cell types in the animal model and in the human

### Example 1: parkinson's disease

In term of complexity the damage present at the level of the *substantia nigra* is in some respects relatively low compared with other neurological disorders; indeed, a substitutional role can be hypothesized for the transplanted cells in this context. The transplantation of cells and stem cells in animal models of PD has been performed for many years. Moreover, a transplantation approach in humans affected with PD has been pursued using fetal tissue from the 5 to the 9th week post conception (Lindvall et al., 1994; Brundin et al., 2000; Kefalopoulou et al., 2014). Although these cells cannot be considered stem cells *per se* since they were not cultivated *in vitro*, the tissue of origin is rich in neural stem cells, so these experiments can be considered to be the precursors of stem cells transplantation in PD.

At the moment at least nine clinical trials are listed in the ClinicalTrials.gov site (U.S. National Institutes of Health), see Table 3. These trials make use of the knowledge gained in animal models, mostly rats (Park et al., 2008; Shetty et al., 2009; Glavaski-Joksimovic et al., 2010; Somoza et al., 2010; Blesa et al., 2012) (Table 3). The PD animal models can be divided into those using environmental or synthetic neurotoxins and those using the *in vivo* expression of PD-related mutations discovered in human patients (genetic). Within the neurotoxic models, compounds that produce both reversible and irreversible outcomes have been used effectively; reserpine is in the former category and the latter category there are 6-hydroxydopamine (6-OHDA), MG-132 (Chung et al., 2007), MPTP (Tieu, 2011), and paraquat and rotenone which were only recently introduced (Blesa et al., 2012). However, recent studies have focused mostly on irreversible toxins to develop PD-related pathology and symptomatology. A typical property of all toxins for PD induction is their capacity to produce an oxidative stress which is most likely responsible for death in dopaminergic neuronal populations which reflects what is seen in PD. Although there are some discrepancies between the time factor in these models and the time factor in the human condition, the value of neurotoxin-based animal models in the study of PD is undeniable (Blesa et al., 2012).

**Table 3:** **Clinical trials using stem cells for the PD treatment**.

**Name of the study location/clinicaltrials.gov identifier**	**Status start and end of the study**	**Number of recruited patients**	**Type of cells/ intervention**	**Study design/primary purpose**	**Outcome measures**	**Preclinical/clinical literature**
Autologous mesenchymal stem cell transplant for Parkinson's disease	November 2011 (final data collection date for primary outcome measure)	5	Autologous bone marrow derived stem cells transplant	Endpoint classification: safety/efficacy study	Primary: improvement in clinical condition of the patient assessed using UPDRS (UNIFIED PARKINSON'S DISEASE RATING SCALE)	Arias-Carrion and Yuan, 2009/no publications associated to the trial
India/NCT00976430				Intervention model: single group assignment		
				Masking: open label		
Mesenchymal stem cells transplantation to patients with Parkinson's disease Cina/NCT01446614	Recruiting	20	Intravenous administration of autologous bone marrow derived mesenchymal stem cells	Endpoint classification: safety/efficacy study	Primary: number of participants with adverse events 1 month after transplantation	Park et al., 2008; Shetty et al., 2009; Glavaski-Joksimovic et al., 2010; Somoza et al., 2010/no publications associated to the trial
	October 2011					
	June 2014			Intervention model: single group assignment		
				Masking: open label	Secondary: effect assessment 1 month after transplantation and later	
				primary purpose: treatment		
Evaluation of safety and tolerability of fetal mesencephalic dopamine neuronal precursor cells for Parkinson's disease Republic of Korea/NCT01860794	Recruiting	15	Evaluation of safety and tolerability of Fetal mesencephalic dopamine neuronal precursor cells as a treatment for patients with Parkinson's disease	Intervention model: single group assignment	Primary: presence or absence of cancer formation and infection within 5 years after transplantation	No publications provided/no publications associated to the trial
	May 2013					
	February 2018			Masking: single blind (outcomes assessor)		
				Primary purpose: treatment	Secondary: score UPDRS) within 5 years after transplantation.	
					Detection of positron emission in Putamen.	
					Dyskinesia	
					Pronation-supination test	
Rajavtihi neuronal adult stem cells project Thailand/NCT00927108	Unknown/July 2009	10	Oligodendrocyte progenitor cell	Basic science	Not described	No publications provided/no publications associated to the trial
	December 2011					
Study to assess the safety and effects of autologous adipose-derived stromal in patients with Parkinson's disease Mexico/NCT01453803	Recruiting/May 2011	10	Autologous adipose-derived stromal cells	Allocation: non-randomized	Primary: presence or absence adverse effects, mesure of UPDRS	No publications provided/no publications associated to the trial
	June 2015			Endpoint classification: safety/efficacy study	Secondary: reduction of Parkinson's medication	
				Intervention model: single group assignment		
				Masking: openlabel primarypurpose: treatment		
Molecular analysis of human neural STEM Cells USA(company) /NCT01329926	Enrolling by invitation/June 2011	20	The aim of this study is to develop and optimize methods to isolate, propagate and differentiate adult human neural stem cells from patients with degenerative neurological disorders like Parkinson's disease	Basic science	Isolation and propagation of adult human neural stem cells from patients with Parkinson's disease	No publications provided/no publications associated to the trial
	June 2014					
Clinical trial to evaluate Bone marrow stem cell Therapy for progressive supranuclear Palsy a rare form of Parkinsonism Italy/NCT01824121	December 2012	25	Mesenchymal stem cells (MSCs) isolated from Bone marrow collected from the iliac crest	Randomized	Primary: incidence of adverse events.	No publications provided/no publications associated to the trial
	December 2014			Endpoint classification: safety/efficacy study: double blind	Secondary: striatal density of dopamine	
				Primary purpose: treatment		
Derivation of induced pluripotent stem cells from somatic cells donated by patients with neurological diseases for the study of the pathogenesis of the disorders and development of novel therapies Israel/NCT00874783	April 2009	120	Human fibroblasts and possibly other human somatic cells reprogrammed.120 donors to cover 10 different neurodegenerative disorders based on 10 donors per disorder and 20 healthy control donors	Basic science Preparation of iPs from people with neurodegenerative pathology to study their biological differences	Not provided	Yu et al., 2007/no publications associated to the trial
	December 2014					
Peripheral blood stem cell collection from adult volunteers USA/NCT00033774	April 2002	Not provided	Bone marrow stem cells collection	Basic science	Not provided	Orkin, 2000; Wei et al., 2000; Lemischka, 2001/no publications associated to the trial
	last update January 2013					

*The Table describes: in row 1 the name of the clinical trial, the location and the ClinicalTrials.gov identifier; in row 2 the Status, the Start and end of the study and the number of recruited patients; in row 3 the type of cells used and the method of administration; in row 4 the study design and the primary purpose; in row 5 the outcomes; in row 6 the preclinical and clinical literature*.

The recent identification of different genetic mutations such as parkin,α-synuclein and others has led to the development of genetic models of PD (Dawson et al., 2010); however, it is important to remember that, at most, only 10% of PD cases are due to genetic mutations (Dauer and Przedborski, 2003), while the majority of PD cases arise from unknown origins.

Other trials, meanwhile, were dedicated to the study of the properties of cells obtained from PD patients (as well as from other pathologies and healthy patients) (Orkin, 2000; Wei et al., 2000; Lemischka, 2001; Yu et al., 2007; Arias-Carrion and Yuan, 2009) (Table 3). These preliminary *in vitro* studies will allow us—in the near future, we hope—to depict the molecular mechanisms of the pathology.

The effectiveness of these trials is not yet known since the results are not yet published (Table 3).

### Example 2: amyotrophic lateral sclerosis

The complexity of ALS make this motor neuron disease very difficult to treat, as is confirmed by the large failure rate of clinical trials: indeed to date more than 30 clinical trials (of conventional drugs) have ended in disappointment. Increasing the odds of success for future clinical trials requires improvements in the preclinical tests. New technical advancements which allow the visualization of sick motor neurons, can bring novel insights. The development of new genetic models has brought new data about ALS and its relationship with other pathologies.

Within the mutations implicated as causative of the Familiar (F) ALS those involving the gene encoding superoxide dismutase 1 (SOD1) deserve a particular mention since they are responsible for about 20% of FALS cases (Carri et al., 2006). Indeed, many different SOD1 mouse and rat models were created, with different characteristics in terms of disease progression (onset and death), and motor performance (Carri et al., 2006). Other mutants of genes that seem to be involved in ALS have been developed, such Vegf δ/δ and Alsin k/oas, and there have been spontaneous mutations such as Dynein (Loa, Cra1) and Wobbler (which arose as the result of a spontaneous mutation at the Institute of Animal Genetics in Edinburgh) (Carri et al., 2006). Recently, mice were developed with the mutation in the genes encoding the TAR DNA-binding protein 43 (Wegorzewska et al., 2009) and FUS/TLS (Hicks et al., 2000).

Very recently, a mouse model was established to research both the C9ORF72 disease mechanism and the possible therapy. When it is available for the scientific community this model will speed up the research on ALS.

Most of the clinical trials ongoing or already concluded make use of the preclinical trials data obtained from animal models (Yu et al., 2007; Dimos et al., 2008; Cho et al., 2010a; Choi et al., 2010b; Karussis et al., 2010; Kim et al., 2010; Blanquer et al., 2012; Koh et al., 2012a,b; Kwon et al., 2012; Robberecht and Philips, 2013) (Table 4). These studies, though, demonstrate that, so far, translation to the human ALS patient is poor. To date, from the 18 clinical trials only two publications have been produced (Glass et al., 2012; Gropp et al., 2012) and only the former really applied to the human patient, while the second concerned the preparation of iPS from cells obtained from patients (Table 4). Feldman and coworkers (Glass et al., 2012) demonstrated the safety of the treatment with Human Spinal Cord Derived Neural Stem Cells obtained from the spinal cord of a 8-week-old fetus, and included testing against many different variables. They have many adverse effects—transient encephalopathy, pulmonary emboli, CSF leak, wound dehiscence, bronchitis/pneumonia, dyspnea, atrial fibrillation, vomiting, basal cell carcinoma—which were most likely related to the injection procedure itself. No rejection markers were detected in the transplanted individuals. On the basis of these results the trial was considered “successful.” Hitherto, since the stated aim of studies was to test safety in an ALS population, very little could be said about effectiveness (Glass et al., 2012). In the work led by Reubinoff (Gropp et al., 2012) a rigorous method of teratoma assay was set up in order to analyze the pluripotency of human ES cells and the biosafety of their differentiated progeny in such a way as to allow a safer translation to humans.

**Table 4:** **Clinical trials using stem cells for the ALS treatment**.

**Name of the study location/clinicaltrials.gov identifier**	**Status start and end of the study**	**Number of recruited patients**	**Type of cells/ intervention**	**Study design/primary purpose**	**Outcome measures**	**Preclinical/clinical literature**
Clinical trial on the use of autologous bone marrow stem cells in amyotrophic lateral sclerosis	Active not recruiting	63	Laminectomy and bone marrow stem cells transplantation	Randomized safety/efficacy study	Primary: forced vital capacity.	No publications provided/no publications associated to the trial
Spain/NCT01254539	October 2010		Intrathecal infusion of autologous bone marrow stem cells	Double blind	Secondary: absence of adverse events; neurophysiological, neuroradiological, and respiratory variables	
	November 2014		Intrathecal infusion of placebo (saline solution)	Primary purpose: treatment		
Dose escalation and safety study of human spinal cord derived neural stem cell transplantation for the treatment of amyotrophic lateral sclerosis	Enrolling by invitation only	18	5 sequential cohorts with 3 subjects in each cohort. Each cohort will follow a dose escalation plan. No control group is included. All patients will received spinal cord injections of HSSC	Safety Study	Primary: safety, toxicity, and maximum tolerated (safe) dose of human spinal cord-derived	No publications provided/Glass et al., 2012
USA/NCT01730716	May 2013			Primary purpose: treatment	Secondary: (1) attenuation of motor function loss; (2) maintenance of respiratory capacity; (3) stabilization of the pathology; (4) reduction of spasticity/rigidity if present; and (5) graft survival at autopsy if and when there is mortality	
	April 2014					
Human spinal cord derived neural stem cell transplantation for the treatment of amyotrophic lateral sclerosis (ALS)	Active not recruiting	18	Transplantation of human spinal cord derived neural stem cell for the treatment of ALS	Safety study	Primary: safety	Robberecht and Philips, 2013/Glass et al., 2012
USA/ NCT01348451	January 2009			Primary purpose: treatment	Secondary: (1) attenuation of motor function loss; (2) changes in muscle performance and pain assessment	
	August 2013					
Clinical trial on the use of autologous bone marrow stem cells in amyotrophic lateral sclerosis	Completed	11	Autologous bone marrow cells collection	Safety/efficacy study	Primary: forced vital capacity	Blanquer et al., 2012/no publications associated to the trial
Spain/NCT00855400	February 2007		Procedure: laminectomy and bone marrow stem cells transplantation	Primary purpose: treatment	Secondary: absence of adverse events	
	February 2010					
The Clinical trial on the use of umbilical cord mesenchymal stem cells in amyotrophic lateral sclerosis	Enrolling by invitation only	30	Heterologous umbilical cord mesenchymal stem cells transplantation	Safety/efficacy study	Primary: forced vital capacity and nerve functional evaluation.	No publications provided/no publications associated to the trial
China/NCT01494480	March 2012			Primary purpose: treatment	Secondary: electrophysiology examination, blood and urinary tests	
	April 2015					
A dose-escalation safety trial for intrathecal autologous mesenchymal stem cell therapy in amyotrophic lateral sclerosis	Recruiting	25	Autologous mesenchymal stem cell transplantation dose escalation	Safety/efficacy study	Primary: number of patients with dose-limiting toxicities	No publications provided/no publications associated to the trial
USA/NCT01609283	May 2012			Primary purpose: treatment	Secondary: adverse effects, blood analysis, development of cancer within 2 years after transplantation	
	May 2014					
Safety study of HLA-haplo matched allogenic bone marrow derived stem cell treatment in amyotrophic lateral sclerosis	Recruiting	18	HLA-haplo matched allogenic bone marrow derived stem cells	Safety/efficacy study	Primary: adverse effects	Choi et al., 2010b; Kim et al., 2010; Koh et al., 2012a,b; Kwon et al., 2012/no publications associated to the trial
Republic of Korea/NCT01758510	December 2012			Primary purpose: treatment	Secondary: motor performance changes	
	June 2014					
Effect of intrathecal administration of hematopoietic stem cells in patients with amyotrophic lateral sclerosis (ALS)	Recruiting	14	Autologous hematopoietic stem cells intrathecal transplantation	Safety/efficacy study	Primary: adverse effects	No publications provided/no publications associated to the trial
Mexico/NCT01933321	December 2012			Primary purpose: treatment		
	January 2014					
Human neural stem cell transplantation in amyotrophic lateral sclerosis (ALS) (hNSCALS)	Recruiting	18	Intra-spinal cord delivery of human neural stem cells in ALS patients	Safety/efficacy study	Primary: safety of a microsurgery human neural stem cells transplantation into spinal cord of ALS patients, percentage of subjects (%) with treatment-related mortality defined as death due to procedure and not to the course of the disease	Robberecht and Philips, 2013/no publications associated to the trial
Italy/NCT01640067	December 2011			Primary purpose: treatment	Number of adverse events related to the procedure	
	September 2016				Changes in neuroradiological and neurophysiological variables	
					Changes in neuropsychological variables	
Safety/efficacy study for the treatment of amyotrophic lateral sclerosis	Ongoing, but not recruiting	6	Infusion of autologous bone marrow-derived stem cells	Safety/efficacy study	Primary: adverse effects	No publications provided/no publications associated to the trial
USA/NCT01082653	March 2010			Primary purpose: diagnostic	Secondary: efficacy	
	December 2013					
Safety and efficacy study of autologous bone marrow derived stem cell treatment in amyotrophic lateral sclerosis	Completed	71	Autologous bone marrow-derived stem cell administered by intrathecal delivery	Safety/efficacy study	Primary: efficacy	Choi et al., 2010b; Kim et al., 2010/no publications associated to the trial
Republic of Korea/NCT01363401	February 2011			Primary purpose: treatment		
	August 2013					
Intraventricular transplantation of mesenchymal stem cell in patients with ALS Islamic	Recruiting	10	Intraventricular injection of bone marrow derived mesenchymal stem cell	Safety/efficacy study	Primary: adverse reaction to the transplantation	No publications provided/no publications associated to the trial
Republic of Iran/NCT01759784	September 2012			Primary purpose: treatment	Secondary: efficacy	
	december 2013					
Intrathecal transplantation of mesenchymal stem cell in patients with als islamic	Recruiting	10	Intratheca injection of bone marrow derived mesenchymal stem cell	Safety/efficacy study	Primary: adverse reaction to the transplantation	No publications provided/no publications associated to the trial
Republic of Iran/NCT01771640	August 2012			Primary purpose: treatment	Secondary: efficacy	
	december 2013					
Creation of a bank of fibroblast from patients with amyotrophic lateral sclerosis: pilot study (ALSCELL)	November 2012	30	The aim of this study is to develop and optimize methods to isolate, propagate and differentiate adult human neural stem cells from patients with ALS	Basic science	The study proposes to investigate the pathophysiology of ALS by setting up a fibroblast bank for studying molecular, cellular and genetic parameters of the pathology.	No publications provided/no publications associated to the trial
France/NCT01639391	August 2014			Preparation of iPs from people with neurodegenerative pathology to study their biological differences	The pathophysiology of ALS will be studied on the 3 types of cells (fibroblasts, iPS, differentiated cells)	
Derivation of induced pluripotent stem cells from an existing collection of human somatic cells	Ongoing, but not recruiting	25	Derivation of induced pluripotent stem cells from an existing collection of human somatic cells	Basic science	Induction of the differentiation from the produced iPS cells obtained from somatic cells. Study of the specific cell lineages and the progeny. This will allow the developing of new therapeutic approaches	Dimos et al., 2008/no publications associated to the trial
Israel/NCT00801333	November 2008			Preparation of iPs from people with neurodegenerative pathology to study their biological differences.		
	December 2014					
Autologous cultured mesenchymal bone marrow stromal cells secreting neurotrophic factors (MSC-NTF), in patients with amyotrophic lateral sclerosis (ALS)	Recruiting	12	Transplantation of escalating doses of autologous cultured mesenchymal bone marrow stromal cells secreting neurotrophic factors (MSC-NTF)	Safety/efficacy study	Primary: safety evaluation and tolerability of a single treatment administration in an escalating-dose of autologous cultured mesenchymal bone marrow stromal cells secreting neurotrophic factors (MSC-NTF)	Karussis et al., 2010/no publications associated to the trial
Israel/NCT01777646	December 2012			Primary purpose: treatment	Secondary: efficacy on progression of the disease, muscle strength and bulk	
	February 2014					
Autologous cultured mesenchymal bone marrow stromal cells secreting neurotrophic factors (MSC-NTF), in patients with amyotrophic lateral sclerosis (ALS)	Completed	12	Transplantation of escalating doses of autologous cultured mesenchymal bone marrow stromal cells secreting neurotrophic factors (MSC-NTF)	Safety/efficacy study	Primary: safety evaluation and tolerability of a single treatment administration in an escalating-dose of autologous cultured mesenchymal bone marrow stromal cells secreting neurotrophic factors (MSC-NTF)	Karussis et al., 2010/no publications associated to the trial
Israel/NCT01051882	June 2011			Primary purpose: treatment	Secondary: efficacy on progression of the disease, muscle strength and bulk	
	March 2013					
Development of iPS from donated somatic cells of patients with neurological diseases	Ongoing, but not recruiting	120	The major goal of the project is to develop human iPS cells from cell cultures from skin biopsies or the patient's hair.	Basic science	Not provided	Yu et al., 2007/Gropp et al., 2012
Israel/NCT00874783	April 2009		Human fibroblasts and possibly other human somatic cells reprogrammed. 120 donors to cover 10 different neurodegenerative disorders based on 10 donors per disorder and 20 healthy control donors	Preparation of iPs from people with neurodegenerative pathology to study their biological differences		
	December 2014					

Rows as in Table 3.

There are probably many reasons why the translation between ALS preclinical-trials and clinical trials is inefficient. The first of these is the timetable of intervention, since in many cases the risks of transplantation (with multiple injections at the spinal cord or brain level) are high and it can only be performed in a patient who is at the later stages of the disease. On this view, an earlier intervention could be more advantageous. A second reason is the type and number of cells that need to be transplanted and here the issue of safety is primary: indeed almost all the trials conducted for ALS included a phase I.

### Example 3: spinal cord injury

Spinal cord injury is a pathological state that consists of at least of two phases: acute and chronic. Although the closest animal model to human SCI is represented by primates their use is limited in many countries, so the most frequently models are rodents: rats and mice. In these models the injury can be performed by, for instance, aortic occlusion (Lang-Lazdunski et al., 2000) or by clip compression (Von Euler et al., 1997). On the other hand, a very widely used model of SCI is the contusive approach where the laminectonize spinal cord is struck, in earlier studies, by a weight (weight drop) (Gale et al., 1985) and in later studies by a cylinder whose dimensions vary according to the type of animal and the region of the spinal cord under study, and with strength, speed and displacement-controlled (Scheff et al., 2003). The injured animal can be studied with regards to behavioral, sensorial and immunohistological factors. An early intervention could avoid the damage caused by the immune-system which is responsible for many detrimental effects, while a late intervention could be indicated if the intention is substitution. Within the 16 clinical trials reported in Table 5, six were dedicated to chronic patients (NCT01393977, NCT01772810, NCT01676441, NCT01873547, NCT01186679, and NCT00816803). The literature used for the submission of these trials includes preclinical studies on mice and rats but also takes note of previous clinical trials (Moviglia et al., 2006; Zurita and Vaquero, 2006; Parr et al., 2007; Geffner et al., 2008; Sheth et al., 2008; Cho et al., 2009; Pal et al., 2009; Paul et al., 2009; Hu et al., 2010; Osaka et al., 2010; Hernandez et al., 2011; Ra et al., 2011; Park et al., 2012). As reported for other pathologies (see Tables 3, 4) very few published works were produced; the only example we are aware of is the work obtained from trial NCT00816803 (El-Kheir et al., 2013). The main outcome of this work was that, in the group of the patients treated with cells, 17 out of 50 managed to show an improvement as measured by the American Spinal Injury Association (ASIA) Impairment Scale (AIS) (for more details see http://www.asia-spinalinjury.org/elearning/ISNCSCI_Exam_Sheet_r4.pdf.), whereas none of 20 controls not treated with cells managed to show any improvement.

**Table 5:** **Clinical trials using stem cells for the SCI treatment**.

**Name of the study location/clinicaltrials.gov identifier**	**Status start and end of the study**	**Number of recruited patients**	**Type of cells/ intervention**	**Study design/primary purpose**	**Outcome measures**	**Preclinical/clinical literature**
Autologous bone marrow stem cell transplantation in patients with spinal cord injury	Status of this study is unknown	20	Patients will undergo autologous bone marrow stem cell transplantation into the lesion area	Non-randomized	Primary: feasibility and safety of bone marrow stem cell transplantation in patients with spinal cord injury	No publications provided/no publications associated to the trial
Brazil/NCT01325103				Safety/efficacy study	Secondary: functional improvement in muscle strength	
				Primary purpose: treatment		
Study the safety and efficacy of bone marrow derived autologous cells for the treatment of spinal cord injury (SCI)	Active recruiting	50	Intrathecal transplantation of autologous stem cell,100 millions per dose in 3 divided doses at interval of 10 days	Safety/efficacy study	Primary: improvement in overall sensory for motor control	No publications provided/no publications associated to the trial
India/NCT01833975	March 2011			Double blind	Secondary: improvement in pain sensation and Significant changes in Muscle Tones from base line.	
	July 2014			Primary purpose: treatment		
Autologous stem cells for spinal cord injury (sci) in children	Active recruiting	10	Bone marrow cell harvest and transplantation are safe in children with spinal cord injury, using pre-transplantation spinal cord as control	Safety/efficacy study	Primary: neurological study by ASIA	No publications provided/no publications associated to the trial
USA/NCT01328860	April 2011			Double blind	Secondary: standard Neuropathic Pain study	
	October 2014			Primary purpose: treatment		
Study of human central nervous system stem cells (HuCNS-SC) in patients with thoracic spinal cord injury	March 2011	12	A Phase I/II study of the safety and preliminary efficacy of intramedullary spinal cord transplantation of human central nervous system stem cells (HuCNS-SC)	Safety/efficacy study	Primary: types and frequencies of adverse events and serious adverse events. Analysis of types and frequencies of adverse events 1 year after transplant	No publications provided/no publications associated to the trial
Switzerland/NCT01321333 (Company)	March 2016			Double blind		
				Primary purpose: treatment		
Autologous mesenchymal stem cells in spinal cord injury (SCI) Patients (MSC-SCI)	Enrolling by invitation	30	Intralesional transplantation of autologous mesenchymal stem cells	Safety/efficacy study	Primary: safety of autologous expanded mesenchymal stem cells transplantation in SCI patients	Moviglia et al., 2006; Pal et al., 2009; Ra et al., 2011/no publications associated to the trial
Chile/NCT01694927	January 2012			Double blind	Secondary: functional improvement in muscle strength; functional improvement in sphincters control; functional improvement in spasticity control	
	June 2014			Primary purpose: treatment		
Difference between rehabilitation therapy and stem cells transplantation in patients with spinal cord injury in China	Recruitment status of this study is unknown	60	Efficacy difference between rehabilitation therapy and umbilical cord derived mesenchymal stem cells transplantation in patients with acute or chronic spinal cord injury in China	Safety/efficacy study	Primary: electromyogram and electroneurophysiologic test	No publications provided/no publications associated to the trial
China /NCT01393977	January 2011			Primary purpose: treatment		
	May 2012					
Safety study of human spinal cord-derived neural stem cell transplantation for the treatment of chronic SCI	August 2013	8	The treatment will consist of laminectomy or laminoplasty of 1–4 vertebral segments overlying the region of spinal cord injury. Six direct injections into spinal parenchyma performed of HSSC will be administered bilaterally (3 on each side of midline): 2 at rostral and caudal edge of the injury site and 1 into approximately one segment length inferior to the injury site	Safety/efficacy study	Primary: determine the safety of human spinal stem cell transplantation for the treatment of paralysis and related symptoms due to chronic spinal cord injury	No publications provided/no publications associated to the trial
USA/NCT01772810 (Company)	February 2014			Primary purpose: treatment	Secondary: the study is to evaluate the graft survival in the transplant site by MRI and the motor and sensory	
To study the safety and efficacy of autologous bone marrow stem cells in patients with spinal cord injury (ABSCI)	Recruiting	15	Autologous bone marrow derived stem cells transplanted intrathecally into patients with spinal cord injury	Safety/efficacy study	Primary: evaluation of the number of participants with adverse events as a measure of safety and tolerability	Geffner et al., 2008/no publications associated to the trial
India/NCT01730183	November 2012			Primary purpose: treatment	Secondary: assessment motor, sensory and sphincteric function	
	November 2014					
Mesenchymal stem cells transplantation to patients with spinal cord injury	Recruiting	20	Intravenous combined with intrathecal administration of autologous bone marrow derived mesenchymal stem cells to patients with spinal cord injury	Safety/efficacy study	Primary: evaluation of the number of participants with adverse events as a measure of safety and tolerability	Zurita and Vaquero, 2006; Parr et al., 2007; Sheth et al., 2008; Cho et al., 2009; Paul et al., 2009; Hu et al., 2010, Osaka et al., 2010; Hernandez et al., 2011/no publications associated to the trial
China/NCT01446640	October 2011			Primary purpose: treatment	Secondary: assessment motor, sensory and sphincteric function	
	June 2014					
Transfer of bone marrow derived stem cells for the treatment of spinal cord injury	Ongoing, but not recruiting participants	10	Infusion bone marrow-derived mesenchymal stem cells into the spinal	Safety/efficacy study	Primary: safety	No publications provided/no publications associated to the trial
USA/NCT01162915 (Company)	July 2010			Primary purpose: treatment		
	December 2013					
Safety and efficacy of autologous mesenchymal stem cells in chronic spinal cord injury	Recruiting	32	A Phase II/III clinical trial to evaluate the safety and efficacy of bone marrow-derived mesenchymal stem cell transplantation in patients with chronic spinal cord injury	Safety/efficacy study	Primary: motor score	Park et al., 2012/no publications associated to the trial
Republic of Korea/NCT01676441	August 2008			Primary purpose: treatment	Secondary: sensory score, motor Evoked potentials, somatosensory evoked potentials	
	December 2014					
Different efficacy between rehabilitation therapy and stem cells transplantation in patients with SCI in China (SCI-III)	Recruiting	300	Different efficacy between rehabilitation therapy and umbilical cord derived mesenchymal stem cells transplantation in patients with chronic spinal cord injury in China	Safety/efficacy study	Primary: neurological function	No publications provided/no publications associated to the trial
China/NCT01873547	June 2012			Primary purpose: treatment	Secondary: electromyogram and electroneurophysiologic test	
	December 2014					
Safety and efficacy of autologous bone marrow stem cells in treating spinal cord injury (ABMST-SCI)	Completed	12	Surgical transplantation of autologous bone marrow stem cells with glial scar resection for patients of chronic spinal cord injury and intra-thecal injection for acute and subacute injury	Safety/efficacy study	Primary: number of Participants with adverse events as a measure of safety and tolerability. Significant clinical improvement in ASIA	No publications provided/no publications associated to the trial
India/NCT01186679	January 2008			Primary purpose: treatment	Secondary: changes in the MRI, Neurological improvement (cranial/spinal reflexes) and evoked potentials study	
	August 2010					
Safety and effect of adipose tissue derived mesenchymal stem cell implantation in patients with spinal cord injury	Recruiting	15	Intravenous injection of autologous adipose derived mesenchymal stem cells. dose: 2 × 10^8^ cells/20 μl intrathecal injection of autologous adipose derived mesenchymal stem cells. dose: 5 × 10^7^ cells/2μl Into a spinal cord injection of autologous adipose derived mesenchymal stem cells. dose: 2 × 10^7^ cells/μl	Safety/efficacy study	Primary: number of participants with adverse events as a measure of safety and tolerability. Significant clinical improvement in ASIA	No publications provided/no publications associated to the trial
Republic of Korea/NCT01769872	January 2013			Primary purpose: treatment	Secondary: MEP/SSEP To evaluate the change of treated spinal cord before cell implantation and at 3 and 6 months post injection of MSCs	
	April 2014					
Intrathecal transplantation of autologous adipose tissue derived msc in the patients with spinal cord injury	Recruiting	15	The effect of intrathecal transplantation of autologous adipose tissue derived mesenchymal stem cells in the patients with spinal cord injury, Phase I clinical study	Safety/efficacy study	Primary: safety and significant MRI change before and after intervention	No publications provided/no publications associated to the trial
Republic of Korea/NCT01624779	April 2012			Primary purpose: treatment	Secondary: significant neurological and electrophysiological function change before and after intervention	
	December 2013					
Cell transplant in spinal cord injury patients	Completed	80	Safety of autologous bone marrow derived cell transplant in chronic spinal cord injury patients at the sites of injury	Safety/efficacy study	Primary: safety of autologous BM transplant	No publications provided/publication related to the trial:El-Kheir et al., 2013
Egypt/NCT00816803	May 2005			Primary purpose: treatment	Secondary: efficacy of BM cell transplant in improving neurological functions in patients with chronic SCI	
	December 2008					

Rows as in Table 3.

Within the cell therapy subgroup of 15 patients with a baseline AIS A (complete lack of motor and sensory function below the level of injury, including the anal area), 2 patients converted to AIS C (some muscle movement is spared below the site of injury, but 50 percent of the muscles caudally to the level of injury cannot move against gravity) and 6 patients improved to AIS B (Some sensation below the level of the injury, including anal sensation). Similarly, within the cell therapy subgroup of 35 patients with a baseline AIS B, 9 patients converted to AIS C. In addition, out of 50 patients treated with cell therapy (AIS A and B), 23 patients had their ASIA motor score increased by ≥10 points. The authors were encouraged by these results and they predicted that a greater number of transplantations and cells per transplantation could perhaps improve the already promising results.

### Example 4: epilepsy

Animal models for this pathology can be divided into two main groups: induced and genetic. The genetic models include animals with spontaneous recurrent seizure: mice or rats, epileptic dogs, transgenic mice, and animals with reflex seizure such as DBA/2 mice, gerbils and photosensitive baboons, while the induced seizure models include the electrically induced and the chemically induced (Loscher, 2011).

Only one trial with the use of stem cells in epileptic patients is indicated as ongoing in clinical trial.gov (Table 6). In this work Bone Marrow Stem Cells are used, and it builds on studies performed on animal models, mostly rats, where different kinds of stem cells—human neural fetal stem cells and neural or embryonic mouse stem cells—were used (Chu et al., 2004a; Ruschenschmidt et al., 2005). In this trial the authors used bone marrow stem cells because of their high availability and their potential role in re-establishing the normal interaction between nerve cells; no data about the effectiveness of the procedure are available yet for this trial. This approach probably does not have any substitutional role vis-a-vis the damaged or malfunctioning cells.

**Table 6:** **Clinical trials using stem cells for the Epilepsy treatment**.

**Name of the study location/clinicaltrials.gov identifier**	**Status start and end of the study**	**Number of recruited patients**	**Type of cells/intervention**	**Study design/primary purpose**	**Outcome measures**	**Preclinical/clinical literature**
Autologous bone marrow stem cells transplantation in patients with temporal lobe epilepsy	Ongoing, but not recruiting participants	20	Transplantations with autologous bone marrow mononuclear stem cells by selective posterior cerebral artery angiography	Non-randomized safety/efficacy study	Primary: seizure frequency	No publications provided/no publications associated to the trial
Brazil/NCT00916266				Primary purpose: treatment	Secondary: adverse effects, hippocampal volume, cognitive performance	

Rows as in Table 3.

### Example 5: stroke

Stroke results either from the rupture of a cerebral blood vessel or from the occlusion of a cerebral artery. As described for other pathologies, rodents such as mice and rats have been extensively used as animal models of stroke, although rabbits, pigs and primates have also been utilized. These models can be divided into two categories: those in which stroke occurs spontaneously and those in which the pathology is induced by the researcher, and this latter type can be either global or focal ischemia.

Global ischemia mimics the cerebral damage that takes place after cardiac arrest and its significance is due to the fact that the incidence of cognitive deficits in all patients that survive sudden cardiac arrest is as high as 50% (Lim et al., 2004). An interesting, widely used model of stroke is middle cerebral artery occlusion (MCAO), produced surgically (Tamura et al., 1981) or by thromboembolic agents that mimic the most common cause of ischemic stroke in humans (Kilic et al., 1998). MCAO in rodents can induce long-term sensorimotor and cognitive deficits and postural and sensory reflexes (Bouet et al., 2007; Freret et al., 2009). Transitory MCAO permits the investigation not only of brain injury associated with ischemia, but also of the cerebral outcomes of reperfusion.

Models of sub-arachnoid hemorrhage (SAH) produce intracranial bleeds in the subarachnoid space between the arachnoid membrane and the pia mater and cause rupture of intracranial vessels that reflects the clinical condition of aneurysmal SAH in humans (Bederson et al., 1995; Veelken et al., 1995). Intracerebral hemorrhage (ICH) can be induced in an animal model by means of the intracerebral injection of bacterial collagenase, which disrupts the basal lamina of blood vessels, causing spontaneous bleeding into the surrounding brain tissue (Maclellan et al., 2008).

Spontaneous stroke models [Spontaneously Hypertensive Stroke-Prone Rat (SHRSP)] were produced as a sub strain of the spontaneously hypertensive rat (SHR). SHRSP rats develop increasing levels of blood pressure from 6 weeks of age, and stroke symptoms by 20 weeks (Bailey et al., 2011). Some transgenic animals (mostly mice) can allow the study of rare forms of stroke as a result of single gene mutations (Markus, 2011).

A variety of cellular approaches have been used for the treatment of stroke. In the nineties, fetal neocortical grafts (Grabowski et al., 1992) and fetal porcine striatal cells (Savitz et al., 2002) were used in transplantation experiments for stroke.

Several types of MSC, ESC, and NSC (Neural progenitors (NP) of human origin) have been used for the treatment of stroke in preclinical trials. For instance, it was demonstrated that human NP were able to induce behavioral improvement 5 weeks after transplantation (Jeong et al., 2003).

Many preclinical treatments of stroke induced by means of MCA occlusion were performed using Human Umbilical Cord Blood (UCB) cells, Bone Marrow Stem Cells (BM-SC) (Borlongan et al., 2011) and MSCs. These reports described various rates of survival and differentiation of the cells, mostly surrounding the ischemic boundary zone (Chen et al., 2001). Better results were obtained by permeabilizing the blood brain barrier (BBB) by means of mannitol in rats subjected to MCA with reduced infarct size, indicating that a permeable BBB is necessary for the mobilization of cells into the brain (Borlongan et al., 2004). This treatment is able to induce a behavioral recovery 2 months after intravenous transplantation (Park et al., 2009), but the mechanism involved is still not clear and it is possible that cell protection is a result of a trophic effect rather than of cell replacement or integration (Chen et al., 2001).

Extensive studies of the therapeutic potential of ES cells for transplantation in stroke indicated that predifferentiate ES cells showed very low contralateral migration and survival (Buhnemann et al., 2006), whereas immature ES cells migrated extensively after contralateral transplantation in a focal ischemic rat model (Hoehn et al., 2002).

More than 60 trials are ongoing or are completed on a stem cell approach for stroke: the completed trials are presented in Table 7. Most of them made use of the data from the previously described preclinical studies. On the basis of these results, many different trials have been carried out, mostly making use of studies of UCB cells, peripheral blood stem cells and MSCs (Mackie and Losordo, 2011). Six trials on stroke patients were accomplished before the start of 2014. Trial NCT00950521 demonstrated that peripheral blood stem cells did not cause serious adverse events during the study period and induced an improvement in three different score tests (Chen et al., 2014). Trial NCT00761982 conducted in Spain demonstrated that Autologous BM-SC transplantation, done between 5 and 9 days after stroke onset, did not cause stroke recurrence or tumor formation during follow-up, but 2 patients had one partial seizure. Unfortunately this treatment did not induce any behavioral improvements 180 days after transplantation (Moniche et al., 2012). Trial NCT01501773 has not yet been summarized in a scientific paper. It is a large study which enrolled 120 patients and attempted to describe the efficacy of the Intravenous transplantation of Autologous Bone Marrow-derived Stem Cells. A pilot study by the same group demonstrated that these cells were safe, but their efficacy cannot yet be established as the study was small and lacked a control group (Prasad et al., 2012).

**Table 7:** **Accomplished Clinical trials using stem cells for the Stroke treatment**.

**Name of the study location/clinicaltrials.gov identifier**	**Status start and end of the study**	**Number of recruited patients**	**Type of cells/intervention**	**Study design/primary purpose**	**Outcome measures**	**Preclinical/clinical literature**
Efficacy study of CD34 stem cell in chronic stroke patients	Completed	30	Autologous peripheral blood CD34 stem cell/ phase II study	Randomized Efficacy study	Primary: NIH-stroke scale (NIHSS)	Mackie and Losordo, 2011/Chen et al., 2014
China/NCT00950521	June 2009			Double blind	Secondary: European stroke scale (ESS)/ European stroke motor subscale (EMS)	
	December 2010			Primary purpose: treatment		
Autologous bone marrow stem cells in middle cerebral artery acute stroke treatment	Completed	20	Autologous bone marrow stem cells/phase II study	Non randomized/safety-efficacy study	Primary: absence of new neurological deficits and adverse effects during the timeframe	Mackie and Losordo, 2011/Moniche et al., 2012
Spain/NCT00761982	September 2008			Double blind	Secondary: improvement in clinical function as assessed by the modified rankin score, barthel scale and NIH stroke scale	
	August 2011			Primary purpose: treatment		
Intravenous autologous bone marrow-derived stem cells therapy for patients with acute ischemic stroke	Completed	120	Intravenous autologous bone marrow-derived stem cells/phase II study	Randomized/ Safety-efficacy study	Primary: barthel index score	No publications provided/Prasad et al., 2012
India/ NCT01501773	October 2008				Secondary: NIHSS score and functional status	
	October 2011					
Safety and efficacy of autologous stem cell therapy in chronic stroke	Completed	30	Autologous bone marrow mononuclear cells/phase 1–2 study	Safety-efficacy study	Primary: functional independence measure	Borlongan, 2011/no publications associated to the trial
India/NCT02065778	December 2008			Double blind		
	June 2014			Primary purpose: treatment		
Study of autologous stem cell transplantation for patients with ischemic stroke	Completed	12	Autologous bone marrow cell transplantation/phase 1–2 study	Non randomized/safety-efficacy study	Primary: absence of new neurological deficits during the procedure and/or in the 4 months follow-up.	No publications provided/Battistella et al., 2011; Rosado-De-Castro et al., 2013
Brazil/NCT00473057	December 2005			Double blind	Secondary: improvement of neurological deficits and in the neuroimaging exams	
	May 2011			Primary purpose: treatment		
Study of human placenta-derived cells (PDA001) to evaluate the safety and effectiveness for patients with ischemic stroke	Completed	Human placenta-derived cells (PDA001) at 3 different doses/phase 1–2 Study		Randomized/safety-efficacy study	Primary: safety- number of participants with adverse events	No publications provided/no publications associated to the trial
USA/ NCT01310114	March 2011			Double blind	Secondary: clinical response defined by a = 1 point decrease from baseline in the Modified Rankin Scale	
	March 2013			Primary purpose: treatment		

Rows as in Table 3.

In a Brazilian study (NCT00473057) completed in May 2011, it was shown that bone marrow cells transplanted into patients a few months after stroke did not induce signs of worsening in the neurological condition. The treatment induced an improvement trend in the National Institutes of Health Stroke Scale score for all the patients but further studies are required to better evaluate the efficacy of this therapy (Battistella et al., 2011; Rosado-De-Castro et al., 2013).

## Concluding remarks

The translation from preclinical experiments in animal models of human neurological pathologies to the treatment of humans is subject to many great difficulties because of the variability of human pathologies. When we consider the use of stem cells for treatment, the level of complexity is further increased by the extreme physiological heterogeneity of these cells and by their responses to the environment.

Since the characteristics of the pathologies cannot be changed, a major advance in stem cell therapy could be achieved by standardizing the preparation, handling and transplantation of these cells. Such improvements are indispensable to an understanding of the potential for effective translation of preclinical trials, and would significantly reduce the variability of the outcomes of clinical trials.

### Conflict of interest statement

The authors declare that the research was conducted in the absence of any commercial or financial relationships that could be construed as a potential conflict of interest.
